# Fractional Carbon Dioxide Laser Improves Vaginal Laxity via Remodeling of Vaginal Tissues in Asian Women

**DOI:** 10.3390/jcm11175201

**Published:** 2022-09-02

**Authors:** Lin Gao, Wei Wen, Yuanli Wang, Zhaoyang Li, Erle Dang, Lei Yu, Chenxi Zhou, Meiheng Lu, Gang Wang

**Affiliations:** 1 Department of Dermatology, Xijing Hospital, Fourth Military Medical University, Xi’an 710032, China; 2 Department of Urology, Shanghai General Hospital, Shanghai Jiao Tong University School of Medicine, No. 85, Wujin Road, Shanghai 200080, China; 3 Department of Urology, Xijing Hospital, Fourth Military Medical University, Xi’an 710032, China

**Keywords:** fractional carbon dioxide laser, vaginal laxity, sexual function, vaginal tactile imaging

## Abstract

Background: Vaginal laxity (VL) is characterized by the relaxing of the vaginal wall that affects the quality of life and sexual function of patients. The current management of VL such as Kegel exercises and topical or systemic hormonal replacement results in unsatisfactory outcomes; thus, novel modalities are needed to improve the efficacy. Vaginal fractional carbon dioxide (CO_2_) laser treatment has shown growing applications for the treatment of VL, but results show nonconformities due to the lack of objective evaluations. In this study, we aimed to validate the clinical efficacy and biophysical benefits of fractional CO_2_ laser treatment for VL patients with the incorporation of objective approaches. Methods: This is a descriptive study without controls. A total of 29 patients were enrolled and treated with two sessions of FemTouch vaginal fractional CO_2_ laser, with a one-month interval between sessions. Both subjective and objective measurements, including female sexual function index (FSFI), vaginal health index score (VHIS), vaginal tactile imaging (VTI), and histology were used to validate the clinical efficacy and biophysical benefits after treatment. Results: The overall FSFI scores and VHIS scores after the first and second treatment sessions were significantly higher than the baseline scores (*p* < 0.01, *n* = 29). VTI measurements showed a significant increase in maximal pressure resistance (kPa) of both the anterior and posterior vaginal walls at a 10–12-month post-treatment visit compared with pre-treatment controls (*p* < 0.001; *n* = 16). Histological examination showed that laser treatment led to increases in the thickness of the stratified squamous epithelium layer and density of connective tissues in the lamina propria. Conclusions: Fractional CO_2_ vaginal laser treatment can improve both vaginal health and sexual function and restore vaginal biomechanical properties by increasing vaginal tissue tightening and improving vaginal tissue integrity in Asian women. Our data support that fractional CO_2_ vaginal laser is a valid treatment modality for VL.

## 1. Introduction

Vaginal laxity (VL) is a condition which involves the relaxing of the vaginal wall and is commonly present in aging women. A variety of risk factors have been shown to be related to the development of VL, including aging, pregnancy, modes of delivery, obesity, menopause-related estrogen deficiency, and physical and psychological distresses [1,2]. VL is generally diagnosed less frequently than its actual occurrence; a cross-sectional survey showed that 48% of females with at least one vaginal delivery have experienced VL [3]. VL can lead to a decrease in sexual confidence, orgasmic experiences, sexual satisfaction, and overall confidence for both females and their partners [1,2]. The conventional management of VL includes Kegel exercises and topical or systemic hormonal replacements [4]. However, low persistence and compliance rates usually lead to poor outcomes with these treatments [5]. Therefore, novel modalities are needed to improve the efficacy.

In the last two decades, energy-based therapies have emerged as alternative noninvasive options for menopausal-related gynecological symptoms, including VL. The integration of fractional laser technology confers the benefits of quick healing and, consequently, short downtime [6], a particularly important factor in the treatment of extremely sensitive vaginal tissues. Gaspar et al. first demonstrated the effectiveness and safety of fractional carbon dioxide (CO_2_) vaginal laser treatment for vaginal atrophy [7]; this finding is supported by accumulated evidence [8,9,10,11,12]. Histological data have also shown that this procedure can significantly restore the physiological integrity of vaginal tissues [13]. However, in available studies, most outcomes have been evaluated according to subjective approaches such as the female sexual function index (FSFI) and the vaginal health index score (VHIS) by patients or physicians [14,15,16]. The lack of sufficient objective data to support the biomechanical and biophysiological remodeling of vaginal tissue after laser treatment has become a clinical barrier that needs to be addressed.

In this study, we attempted to use both subjective evaluations, including FSFI and VHIS, and objective measurements, such as vaginal tactile imager (VTI) and histology, to validate the clinical efficacy and biophysical benefits of fractional CO_2_ laser treatment for patients with VL. Our results showed that both vaginal health and sexual function have been improved in patients in our cohort. Our data demonstrates the superiority of fractional CO_2_ vaginal laser treatment to the traditional management approaches by the following evidence: (1) it is noninvasive and efficient; (2) it results in a rapid healing process with short downtime; (3) it effectively rejuvenates vaginal tissues by restoring vaginal physiological properties; (4) it remodels the biomechanical features of vaginal tissues; and, (5) it improves the sexual functions of patients.

## 2. Materials and Methods

### 2.1. Study Population

A total of 30 subjects with VL visiting the Department of Dermatology at Xijing Hospital between October 2019 and November 2020 were enrolled in this study. The study was approved by the ethics committee at Xijing Hospital on 18 July 2019 (Approval Number: KY20192082-F-1). This study was registered on ClinicalTrials.gov (accessed on 1 August 2022) (ID: NCT04492176). Risk factors and potential contraindications related to using this treatment were fully discussed with patients before written informed consent was sought and obtained.

A comprehensive physical and sexual function evaluation was carried out for all subjects prior to treatment. VHIS was used for gynecological evaluation. VHIS included five parameters: elasticity, fluid volume, pH, moisture, and epithelial integrity. The scores for each parameter range were from 1 to 5. The FSFI questionnaire with a total of 19 questions was used to assess sexual function: scores were in the range 2–36. VHIS and FSFI were taken for all patients before treatment and one month after each treatment session.

Exclusion criteria included pregnancy; active bacteria, fungal or viral genital infection; use of photosensitizing or anticoagulation agents; receiving pelvic floor physiotherapy, local estrogen, or other vaginal-tightening modalities; cervical dysplasia or cancer, cerebrovascular and thromboembolic events; and a diagnosis of urinary incontinence.

### 2.2. Laser Treatment

Patients were treated with a fractional CO_2_ vaginal laser using the FemTouch probe (AcuPulse, Lumenis, Yokneam, Israel). For endovaginal treatment mode, the laser probe was fully inserted into the top of the vaginal canal, and the laser was fired with an energy fluence of 10 mJ and a spot density of 10–15%. The probe was then rotated 60 degrees, and the laser emitted another impulse; the procedure was repeated until it covered the entire vaginal wall. Then, the probe was withdrawn by one gradation marked on the probe body, and the process was repeated. A total of 3–4 laser passes were delivered along the entire vaginal channel. The tightening of the vaginal introitus and the vulvar zone was carried out with the same laser parameters, except for a lower energy fluence of 7.5–10 mJ, depending on the patient’s tolerance level. All patients received two treatment sessions at a one-month interval.

### 2.3. VTI Measurement

**Figure 1:**
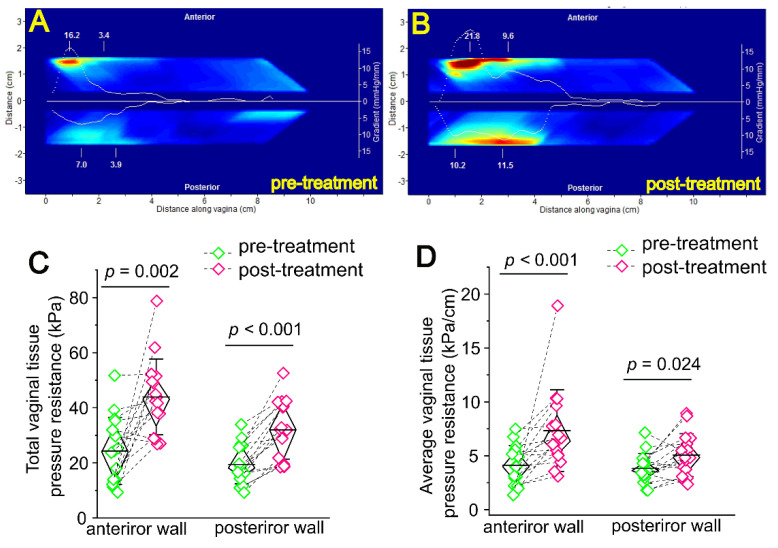
Fractional CO_2_ laser treatment improves vaginal tissue tightening in patients with VL. (**A**,**B**), Tactile images showing pressure gradients for VTI probe insertion for a 42 year old female subject pre-treatment and 12 months post-treatment. A pressure resistance curve (white) was generated during each measurement and displayed in the VIT image. (**C**) Quantitative analysis of total vaginal tissue pressure resistance of the anterior and posterior vaginal walls (kPa). The total area under the pressure curve was computed to determine the maximal pressure, e.g., total vaginal tissue pressure resistance, in both anterior and posterior vaginal walls. (**D**) The average vaginal tissue pressure resistance crossing anterior and posterior vaginal walls was determined by the ratio of total vaginal tissue pressure resistance to the length of the vaginal wall with detectable pressure in the curve (kPa/cm). Dotted lines connect VTI data points before and after treatment for the same subject; diamond boxes represent median (IQR), and whiskers represent SD. Statistical analysis was performed using a Wilcoxon signed-rank test (two-sided).

Changes in the biomechanical properties of vaginal tissues were assessed using a VTI system (Advanced Tactile Imaging, Trenton, NJ, USA) before treatment and 10–12 months after the final laser treatment. The insertion and rotation of the VTI probe deformed the vaginal wall, resulting in integral resistance from the left and right sides of the vaginal tissue, which could be measured by pressure sensors (kPa) on the angled probe of the VTI system and reflect the average vaginal tissue tightening [17,18]. All 30 subjects had VTI measurements prior to treatment but only 16 had VTI measurements at a 10–12-month follow-up visit. The data from these 16 patients were included in the final analysis. A pressure resistance curve was generated during each measurement and is displayed in the VTI image. The total area under the pressure curve was analyzed using the Image J software to determine the maximal pressure, e.g., total vaginal tissue pressure resistance, in both anterior and posterior vaginal walls. The average vaginal tissue pressure resistance was determined by total vaginal tissue pressure resistance/the length of the vaginal wall with detectable pressure in the curve (kPa/cm). For both the anterior and posterior walls, a pressure curve was generated during each measurement (Figure 1A,B). The measurements from the anterior compartment represented tactile responses from pubic bone and urethra, while the measurements from the posterior compartment represented tactile responses from the perineal body and levator ani muscles [18].

### 2.4. Histology Analysis

Punch biopsies (*n* = 2 subjects) were taken before treatment and one month after the final treatment session. The tissues were fixed, embedded, and prepared for routine hematoxylin and eosin staining for histological evaluation. The total area and length of the stratified squamous epithelium layer from each section were determined using ImageJ software. The thickness of the stratified squamous epithelium layer was calculated by the total area/length from each section. An average thickness of the stratified squamous epithelium layer was obtained in five sections from the same sample.

### 2.5. Statistical Analysis

The statistics software SPSS (version 19.0) (IBM Corp, Armonk, NY, USA) was used for statistical analysis. Data are presented as median (interquartile range (IQR)) or mean (SD). A Wilcoxon signed-rank test (two-sided) was used to compare variables of VTI for patients at baseline and after treatment. A Mann–Whitney U test was used to compare differences in FSFI and VHIS between the two groups. A value of *p* < 0.05 was considered statistically significant.

## 3. Results

A total of 30 subjects with VL were enrolled in the study. One patient did not complete the study and was excluded from the final analysis. Demographic and clinical information are listed in Table 1. The average age of the subjects was 37.2 ± 7.8 years old.

FSFI scores after the first and second treatment sessions were 27.0 ± 5.5 and 26.6 ± 4.9, respectively; these scores were significantly higher than baseline scores (24.3 ± 5.2, *p* < 0.01, Table 2). Specific component scores (desire, arousal, lubrication, and satisfaction) showed significant increases after both treatment sessions compared with before treatment (Table 2). The score for the orgasm component showed significant improvement after the first treatment session, but not after the second treatment session, compared with baseline scores (Table 2). The overall VHIS scores after both treatment sessions (first, 19.9 ± 3.6; second, 21.9 ± 3.5) were significantly higher than baseline scores (17.4 ± 3.9, *p* < 0.05, Table 2). There were significant improvements in specific domain scores (vaginal elasticity, secretion, pH, hydration, and epithelial mucous membrane) after both treatment sessions compared with before treatment (Table 2). In addition, the lubrication domain of FSFI, overall VHIS scores, and the vaginal elasticity and hydration domains of VHIS were significantly higher after the second treatment session than after the first treatment session (*p* < 0.05, Table 2).

VTI was used to measure the maximum resistance (kPa) from the vaginal anterior and posterior walls during probe ration; that is, vaginal tightening. Tactile images from VTI before treatment and at 10–12 months post-treatment are shown in Figure 1A,B. The maximal pressure resistance (kPa) of both the anterior and posterior vaginal walls after treatment were significantly higher than the pre-treatment baseline: anterior, 23.86 (13.43, 34.36) versus 42.99 (31.03, 50.83), *p* = 0.002; posterior, 18.33 (14.80, 26.26) versus 32.27 (20.30, 41.90), *p* < 0.001 (Figure 1C). The average pressure resistance (kPa/cm) crossing both the anterior and posterior vaginal walls after treatment were significantly higher than the pre-treatment baseline: anterior, 3.98 (2.40, 5.50) versus 6.34 (5.05, 9.19); posterior, 3.69 (3.10, 4.52) versus 4.88 (3.32, 6.51); *p* = 0.025 (Figure 1D).

**Figure 2:**
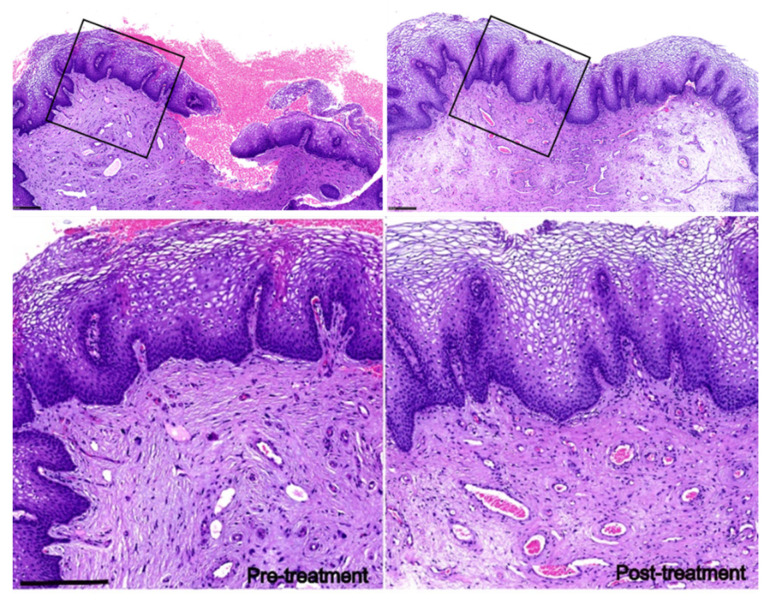
Laser treatment improves vaginal tissue physiological properties in patients with VL. H&E staining of vaginal biopsies taken from subject 1 (50 years old) before and one month after treatment. Scale bar: 250 µm.

Finally, a histological analysis was performed to verify the cellular remodeling of vaginal tissues. Vaginal biopsy tissues were taken from two subjects before and at one month after the final treatment. Both subjects were 50 years old with pregnancy/delivery histories and self-described VL. The laser treatment led to a thicker stratified squamous epithelium layer in both subjects (subject 1, 349.4 ± 54.6 versus 654.8 ± 61.4; subject 2, 401.4 ± 34.5 versus 587.8 ± 34.6 µm, Figure 2). In addition, angiogenesis was more evident and connective tissues in the lamina propria were denser.

No adverse events (e.g., infection, scarring, and bleeding) were reported during the treatment sessions among patients.

## 4. Discussion

In this study, we confirmed that fractional CO_2_ laser treatment can effectively remodel loose vaginal tissues in Asian women by increasing vaginal tightening and physiological integrity. We also demonstrated improvements in both vaginal health and sexual function after treatment. Therefore, this study provided a comprehensive evaluation of the clinical efficacy of fractional CO_2_ laser treatment for VL in Asian women.

Two types of laser systems have been applied for vaginoplasty; that is, CO_2_ (10,600 nm) and Er:YAG (2940 nm) lasers [7,8,19]. The wavelengths of both systems have high water absorption. The development of fractional laser technology, which splits a laser beam into multiple microbeams in order to generate micro-ablative columns (MACs) in tissue [6], created an effective modality for restoring vaginal muscle tone. In 2011, Gaspar et al. were the first to demonstrate the clinical efficacy of fractional CO_2_ vaginal laser treatment for vaginal atrophy [7]; this was followed by several reports presenting similar results for vaginal tissue restoration using both laser systems [8,9,10,11,12]. Histological studies have shown that fractional CO_2_ laser treatment can lead to the restoration of vaginal mucosal structure and related physiological trophism in patients [13], as well as in ex vivo animal models [11,20], which is in line with our histological data. Consistent with this literature, our study showed the clinical efficacy of laser-assisted tightening and the physiological integrity of vaginal tissues in a Chinese population, demonstrating the evolution of this modality into a standard “non-surgical” and “nonhormonal” treatment for women with VL.

A large body of accumulated evidence for the clinical efficacy of laser treatment is based on subjective evaluations, such as FSFI and VHIS, by either patients or physicians [14,15,16]. This indirect evidence, suggesting vaginal tissue and pelvic floor structural remodeling following laser treatment, is crucial for initial assessments but insufficient for further mechanistic studies. Therefore, additional objective validation of changes in the biophysical properties of vaginal tissue is required to provide more evidence-based support for this modality; this type of evidence is scare in the current literature. Our study aimed to overcome this limitation by adding an objective approach such as VTI for a more comprehensive evaluation of treatment efficacy. VTI technology can reveal fine biomechanical changes in vaginal tissues after treatment [18,21]. Lauterbach et al. showed improvement in four biomechanical parameters of vaginal tissue after fractional CO_2_ laser treatment, including vaginal elasticity, tightening, contraction strength of pelvic muscles, and reflex of pelvic muscle contraction [21]. We have demonstrated the restoration of vaginal tightening at both the anterior and posterior vaginal walls using VTI, which is consistent with the report by Lauterbach et al. [21]. These data together demonstrate that VTI provides an objective and quantitative assessment to guide physicians in the use of energy-based therapies.

Applying fractional CO_2_ laser for the treatment of vulvovaginal atrophy has been introduced worldwide and rapidly promoted as both safe and efficacious. Besides VL, several observational studies have reported that CO_2_ laser therapy demonstrates an improvement or cure of symptoms in patients with GSM or SUI [22,23]. Our and other histological studies have revealed that CO_2_ laser treatment restores most vaginal functions, including secretion, absorption, elasticity and lubrication, and the thickness of the vaginal epithelium [24], suggesting that it can be a modality for the improvement of sexual life in patients with post-radiotherapy atrophy or contraindications to estrogen therapy.

There were several limitations to this study. First, the patient cohort was relatively small, and it lacked a sham control. Second, VTI measurements were taken only for a portion of the patients at a 10–12-month follow-up visit due to the lockdown for the COVID-19 pandemic; this may have caused a biased representation of these data for the cohort. Third, VTI parameters for evaluating the strength of pelvic floor muscle contraction are absent. Fourth, the long-term efficacy of laser treatment and the associated quantitative changes in vaginal tissues are absent. A long-term follow-up study will be our next focus. Fifth, histological examinations were carried out in only two patients; the biomarkers related to collagenases and angiogenesis are yet to be explored in response to laser treatment.

## 5. Conclusions

In this study, using subjective evaluations (FSFI and VHIS) in combination with objective VTI measurements and histology, we confirmed the clinical efficacy of fractional CO_2_ laser treatment for Asian patients with VL; this included improvement in both vaginal health and sexual function and a reduction in vaginal laxity by increasing the tightening and physiological integrity of vaginal tissues.

## Figures and Tables

**Table 1:** Demographic and Clinical Information of the Subjects.

Patients, No.	29
Average age (years) (mean ± SD)	37.2 ± 7.8
Postmenopausal Women, No.	3
Nulliparous Women, No.	9
Natural labor/cesarean birth, No.	14/6

**Table 2:** Total and Domain-Specific Scores of FSFI and VHIS in patients Before and After Each Laser Treatment Session.

	Before Treatment	One-Month Post-Treatment	
		First Treatment	Second Treatment
FSFI (*n* = 29)	24.3 ± 5.2	27.0 ± 5.5 **	26.6 ± 4.9 **
Desire	3.2 ± 1.1	3.5 ± 1.2 *	3.6 ± 0.9 *
Arousal	3.9 ± 1.1	4.3 ± 1.0 *	4.2 ± 1.0 *
Lubrication	4.6 ± 1.1	5.1 ± 0.7 **	5.3 ± 0.5 **^,^***
Orgasm	4.1 ± 1.3	4.4 ± 1.1 *	4.2 ± 1.2
Satisfaction	4.0 ± 1.1	4.6 ± 0.9 *	4.7 ± 1.6 *
Pain	4.6 ± 1.2	5.1 ± 1.2 *	4.8 ± 1.1
VHIS (*n* = 29)	17.4 ± 3.9	19.9 ± 3.6 *	21.9 ± 3.5 *^,^***
Vaginal elasticity	3.7 ± 0.9	4.1 ± 0.7 *	4.3 ± 0.6 *^,^***
Vaginal secretions	3.3 ± 1.2	3.9 ± 1.1 **	3.6 ± 1.0 *
pH	2.5 ± 1.2	3.1 ± 1.3 **	2.7 ± 1.0 *
Vaginal mucosa	4.3 ± 0.9	4.5 ± 0.7 *	4.5 ± 0.9 *
Vaginal hydration	3.7 ± 1.0	4.4 ± 0.7 **	4.6 ± 0.6 **^,^***

* *p* < 0.05, ** *p* < 0.01 as compared scores after each treatment session with the baseline scores. *** *p* < 0.05 as compared scores after the second treatment session with the scores after the first treatment session. A Mann–Whitney U test was used to compare the differences between the two groups. Data are presented as mean ± SD.

## Data Availability

Data available on request.
